# A conceptual space for EEG-based brain-computer interfaces

**DOI:** 10.1371/journal.pone.0210145

**Published:** 2019-01-03

**Authors:** Nataliya Kosmyna, Anatole Lécuyer

**Affiliations:** 1 MIT Media Lab, Cambridge, MA, United States of America; 2 Inria Rennes, Rennes, France; Duke University, UNITED STATES

## Abstract

Brain-Computer Interfaces (BCIs) have become more and more popular these last years. Researchers use this technology for several types of applications, including attention and workload measures but also for the direct control of objects by the means of BCIs. In this work we present a first, multidimensional feature space for EEG-based BCI applications to help practitioners to characterize, compare and design systems, which use EEG-based BCIs. Our feature space contains 4 axes and 9 sub-axes and consists of 41 options in total as well as their different combinations. We presented the axes of our feature space and we positioned our feature space regarding the existing BCI and HCI taxonomies and we showed how our work integrates the past works, and/or complements them.

## Introduction

There have been many research works devoted to Brain-Computer Interfaces (BCIs) in the domain of Human-Computer Interaction (HCI). The studies include measuring the attention level of the user [1], the workload [2; 3; 4]. BCIs, have, for example, been used for activity recognition [5], to explicitly interact with applications [6] and games as well as to control the movement of real objects [7]. Combining BCIs with the additional sensors such as eye-tracking [8], gyroscope [9] added more degrees of freedom for the user (e.g. selection of the object is done via eye-tracking and the command is performed using BCIs).

**Fig 1:**
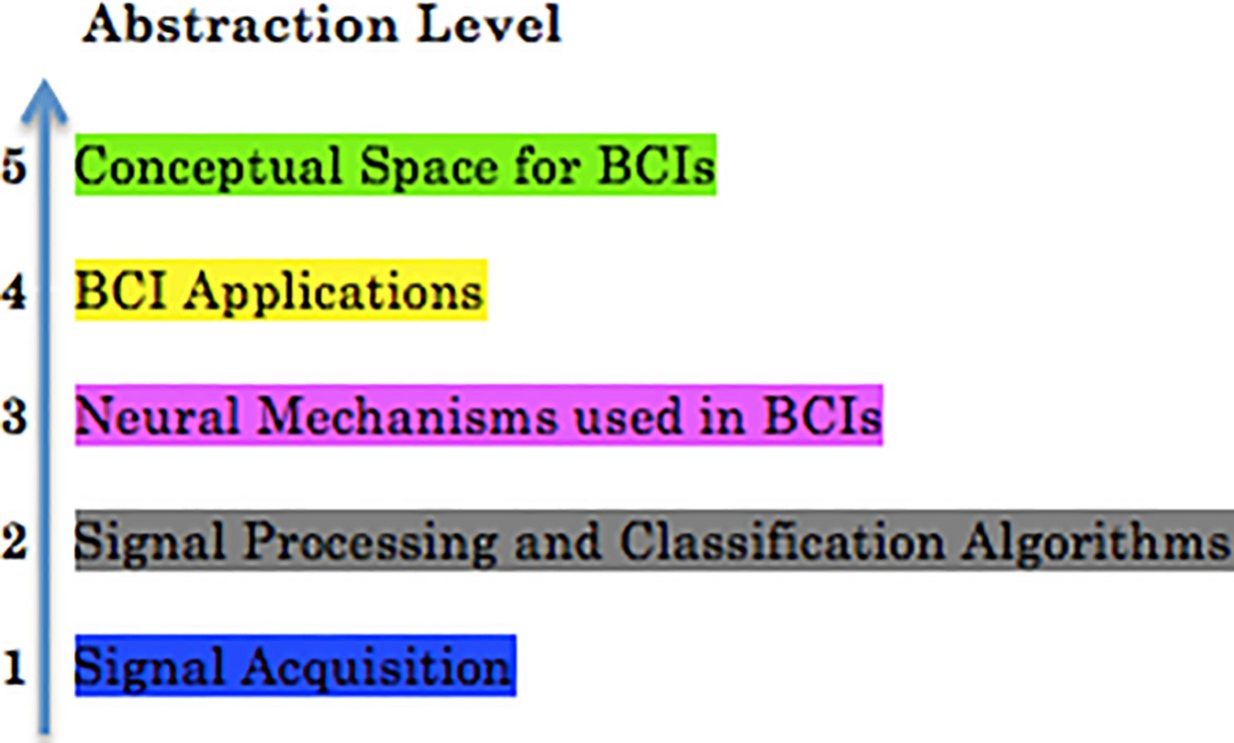
Different levels of abstractions to study BCIs. In green is highlighted the main contribution of our paper. We, however, will cover briefly abstraction levels 1 to 4 in Section 2 of this paper. We introduce the color code, so that the reader can follow the structure of the paper and deside for himself/herself if he/she would like to skip a subsection (a BCI specialist could go though the Section 2 really fast or skip it completely as he/she already has the necessary knowledge).

The domain of Brain-Computer Interfaces (BCIs) itself has emerged and motivated different studies on different levels of abstraction (Fig 1), either related to hardware development (Fig 1, level 1), to the signal processing and classification algorithms (Fig 1, level 2), studying the underlying neural mechanisms (Fig 1, level 3) or the actual applications which use BCIs as an interaction modality (Fig 1, level 4).

However, despite all this technological progress, the adoption of BCIs as an input modality is still quite moderate aside from more « proof-of-concept » applications. We argue that it is in part related to the fact that the BCIs and its commands are not self-revealing [10]: both users and designers should know which commands are available and how to trigger them.

There is currently a gap as no design space on BCIs exists that would help the users, designers and researchers to better understand, compare and reason about the appropriate solutions the BCIs could provide to them. The challenge is to propose a design space, which would be simple to use by the researchers and users who may not be familiar with the full state of the art on BCIs. Our goal is to provide a useful and general framework for defining the conceptual space that could be used for comparing works in this space, and to generalize and unify the preceding proposed classifications.

To address this issue we decide to inspire ourselves from the taxonomies that already exist in HCI by proposing a conceptual space that has descriptive, comparative and generative objectives. This space will:

- Group the existing interaction techniques for BCIs
- Facilitate their comparison
- Facilitate the generation of new techniques.

We introduce a conceptual space, as taxonomy of different features used in EEG-based BCI applications.

To demonstrate the coverage of the conceptual space, we have classified 40 existing BCI systems. The analysis of our conceptual space *per se* reveals directions for future research.

Our manuscript is organized as follows: although the main contribution of this work is a design space, we begin our paper by defining what BCIs are and provide examples of the great variety of systems that use BCIs. This analysis is done to clarify the terminology for the researchers and users who will read this paper. We also provide a short summary in the beginning and in the end of each section. We then present our conceptual space for BCI applications. We discuss future directions for research in the form of case scenarios that our conceptual space has supported. Our expectation is to propose new insights for facilitating the design of novel BCI systems and to further elicit their use and acceptance by the broader public.

## BCI fundamentals

We address this paper to both HCI researchers who are interested in using BCIs in their projects and to BCI researchers who want to further expand the work. For this reason our conceptual space rests on the notions both from HCI and BCI. In order to facilitate the reading of this document, we introduce in the following section the necessary background on BCIs to better understand the choices behind our design space. This is not an extensive state of the art about BCIs (for example, invasive BCIs are not mentioned at all in this paper), but this section provides the necessary basics on BCIs in order to better understand their advantages and drawbacks.

### Defining BCIs. BCI vocabulary

Here is a broad definition of Brain–Computer Interfaces: “BCIs allow capturing the brain activity of users by processing and/or classifying their brain signals with the purpose of controlling any system”. A *crucial* component for BCIs is ability of users to produce stable brain signal patterns in order to facilitate their recognition. Here, we define this skill as “BCI *training/learning”*. *BCI training* could require days of training and repetitive practice.

**Fig 2:**
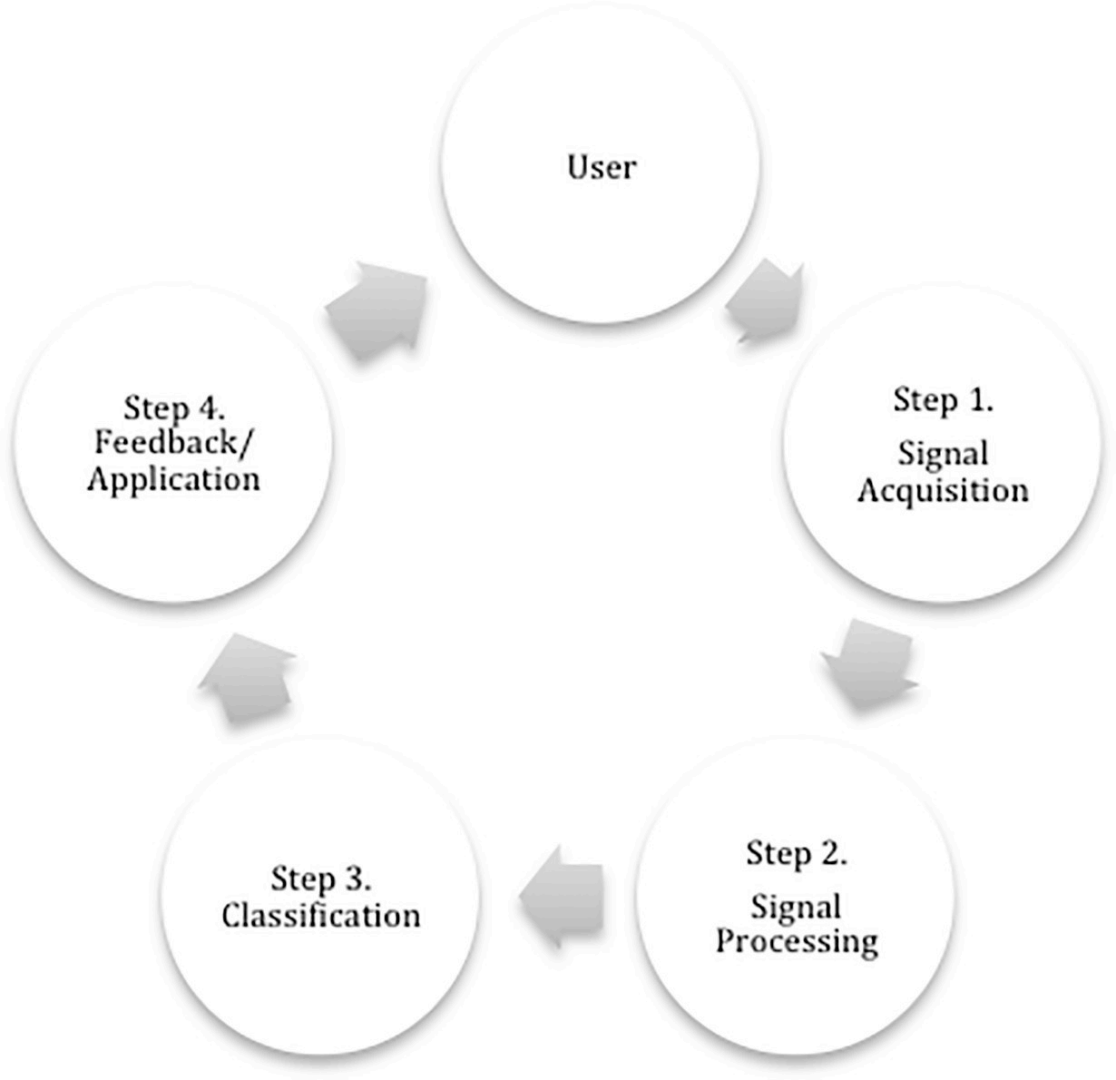
Common BCI loop.

BCI systems nowadays are mostly designed to be a “closed loop between a user and the system. Generally, the user interacts with the system and the system gives feedback about its state after the interaction” [10]. However, a BCI system can also be an open loop, where a user is unaware of the way the system uses their recorded brain activity [11]. The notion of BCI loop was first introduced by [12] and contains the following main steps (Fig 2): signal acquisition (recording of brain activity); signal processing (to remove artefacts); classification (to identify the control signal); feedback/application (to provide information on the outcome of the command and/or brain activity). The research in BCIs nowadays is focused over all steps of this loop. The steps of the loop correspond to different levels of abstractions to study BCIs we introduced on Fig 1. We now review these abstractions levels.

### Abstraction level 1: Signal acquisition

In this subsection we show, that there are several different mechanisms to measure the brain activity of the person, and each of them has the advantages and the drawbacks.

Signal acquisition is based either on invasive or non-invasive methods. Invasive acquisition methods imply opening up or drilling the cranium in order to insert sensors that measure the electrical activity of a restricted area directly on the brain. One of the most common invasive acquisition techniques is Electrocorticography (ECoG), where electrodes are implanted in the desired cortex of the brain where the activity occurs [13].

In contrast, non-invasive methods rely on measuring brain activity by placing sensors on the scalp or around the head without the need of surgical intervention. Non-invasive modalities are more sensitive to noise and measure an attenuated signal compared to invasive acquisition methods. We briefly review some of the main non-invasive acquisition modalities.

#### Electroencephalography (EEG)

EEG is the most commonly used technique to measure this activity: electrodes are placed on the scalp and can be used in pairs to measure the electrical differential. The EEG technique is one of the most studied non-invasive interfaces as it has a fine temporal resolution. The main advantages of EEG lie in the relatively low setup price, possibility of portability and relative ease of use. Among the main drawbacks of EEG is the quality of spatial resolution that tends to be poor. With scalp EEG, the activity recorded by one channel corresponds to the averaged activity of millions of neurons. Only certain types of activity in the superficial layers of the brain can be measured and the amplitude of the electrical activity is in the microvolts. The signal measure for each channel is hooked to an amplifier that amplifies the signals 1000 to 100,000 fold. In a digital acquisition system the analog signal is digitized and typically sampled at a rate of 256 to 512 Hz and then filtered to remove undesirable frequencies (high frequencies correspond to motor noise, low frequencies contain artifacts from heart activity, 50-60Hz signals correspond to artifacts at the frequency of alternative current, the artifacts will be discussed later in the section).

#### Functional Magnetic Resonance Imaging (fMRI)

Functional Magnetic Resonance Imaging (fMRI) is an imaging technique to visualize internal structures of the brain through nuclear magnetic resonance. Functional MRI (fMRI) is a type of MRI scan that measures the hemodynamic response (change in the ratio of oxyhemoglobin and deoxyhemoglobin) related to neural activity in the brain [14]. A limitation of this approach is that it has a low temporal resolution (up to 8 seconds) because the inflow of blood is not an immediate phenomenon. The development of real time fMRI has allowed fMRI to be used to build BCI systems and to produce neurofeedback, the latter being the more popular application given the low temporal resolution of fMRI. We refer the reader to [15] for an exhaustive review of fMRI applications to BCIs and neurofeedback.

#### Functional Near-Infrared Spectroscopy (fNIRS)

fNIRS measures the relative concentration of oxyhaemoglobin and deoxyhaemoglobin by emitting near-infrared photos from an emitter placed on the skull and by measuring the spectrum shift of the photons that cross through a few centimeters away. Compared to other modalities that measure blood oxygenation levels (fMRI), fNIRS has a relatively higher temporal resolution (10Hz vs. 0.5Hz for fMRI). Thus fNIRS allows building BCIs with a reasonable latency between the stimulation onset and the classification output. Moreover, another advantage is that fNIRS is not affected by artifacts from muscle activity and allows to produce BCIs that function in an ambulatory setting [16]. All these advantages and the development of portable fNIRS sensors have prompted significant recent work on developing fNIRS BCIs, especially in HCI, where the ability to move is essential. However, fNIRS is also limited: it has a shallow penetration of no more than 2 cm on average and it is affected by skull thickness and noise from the activity of the vascular system. Thus, fNIRS BCIs also suffer from signal to noise ratio problems.

#### Magnetoencephalography (MEG)

Magnetoencephalography or MEG captures brain activity through measurements of the magnetic fields created by the natural electric currents generated in the brain through sensitive magnetometers [17]. The most common types of sensors are called Superconducting Quantum Interference Devices (SQUID) as they are extremely compact and allow the reduction of the size of the acquisition devices. The brain generates the magnetic fields that are very faint compared to ambient electromagnetic fields and thus shielding is critical. Although sensors are compact, the shielding requires large enclosing bodies and requires users’ heads to be immobile (although movement elsewhere is not an issue). MEG has a greater spatial resolution than EEG and promises to offer high temporal resolutions (~1ms per sample). MEG is mostly used to study neurocognitive phenomena (e.g. the study of feature processing [18], but successful BCIs have been developed as well [19].

#### Positron Emission Tomography (PET)

Positron Emission Tomography (PET) measures the spatial distribution and movement of a radioactive chemical (“tracer”) injected into the tissue of a human. PET scans are expensive (see Table 1). Another disadvantage for using PET lies in the fact that it involves injection of a radioactive material into the blood. The other problem is that although PET, similarly to fMRI has excellent spatial resolution, the delay between the shift in activity and the resulting change in blood flow is between 6 and 9 seconds (one can interpret where the event occurred but not be sure of exactly when it occurred, which makes statements about the relative order of activations problematic) [20].

**Table 1:** Summary of the state of the art on acquisition techniques for BCIs.

Neuroimaging tool	Spatial Resolution	Temporal Resolution	Portability	Price, starting from ($)	Particular remarks
**EEG**	Low	~0.05s	yes	1000	Require motionless, use of gel in some cases, electronic devices could interfere if nearby
**PET**	Very high	~1s	no	125000	Laying down in the scanner, ingestion of hazardous material
**(f)NIRS**	High	~1s	yes	10000	Slow
**fMRI**	High	~1s	no	500000	Laying down in the scanner, complete motionless, exposes subjects to loud noises, no computer usage next to the scanner
**MEG**	High	~0.05s	no	100000	No computer usage next to the scanner

### Summary of the state of the art on acquisition techniques for BCIs

As we can conclude from Table 1, that wraps up this subsection, 3/5 neuroimaging tools described in this section, place restrictions on the user, PET, MEG and FMRI require sitting/laying down in the scanner, which may not be realistic for some human-computer interaction settings. Most of these acquisition tools are quite expensive, and not portable, which also limits the interaction and may not be suited for some HCI applications (depends on the context of use). In our work we will mainly *focus on the description of EEG-based BCI systems* as the most affordable ones for most research labs and centers nowadays.

### Abstraction Level 2: Aspects related to signal processing and classification

**Why this subsection is interesting for HCI researchers?** In this subsection we explain, that in BCIs the commands are not always issued by the person at his/her will, but are very often system-paced, e.g., imposed by the system. This property of BCIs may require particular design choices when proposing an interaction based on BCIs.

#### Classification of brain states

Brain signals are very variable both between people and within a single person. This is due to the low spatial resolution of scalp EEG and to the fact that the measurement of EEG is indirect. This means that averaging is required to obtain reliable measurements, which in turn slows down the speed at which EEG can be processed and used to detect phenomena and results in a low bitrate. The bitrate represents the amount of information transmitted by the BCI per minute. In most cases this is the number of actions performed per minute. The person, who wants to use a BCI-based system should be trained to produce the stable brain patterns The more training the user undergoes or the more training trials are captured, the faster the detection can be made [21]. This training can require hours and days of repetitive practice. Nowadays, we distinguish *synchronous* and *asynchronous* systems.

In *synchronous* (or system-paced) BCIs the commands are imposed by the system. These systems include two stages. *First* stage requires the subject to perform mental tasks (e.g. imagination of tongue movement), in order for the system to collect a sufficient amount of supervised data. At this stage no feedback is provided to the user. The acquired data is processed offline, and allows defining features, classifiers and their parameters. Once the classification accuracy is sufficient from the offline learning, a *second*, supervised stage is proposed: the user is cued on the tasks to perform and now receives feedback on the result of the classification.

In contrast, in *asynchronous* (self-paced) BCIs the commands are issued at any time whenever the user decides. This helps in achieving real-time systems [22], though the training examples are also required for these systems.

Still, the synchronous setting remains common, as the performance of such BCIs can easily be evaluated, thus making this setting desirable for experiments and for comparing system in an in-the-lab setting. Moreover, a continuous classification (required for asynchronous systems) greatly increases the computational requirements towards achieving a real-time BCI system.

Training in BCIs takes from 10 minutes to several hours (at most). However, compared to the inexistent training required for tactile tablets or keyboard based interactions, training a BCI system is a tedious process.

Table 2 summarizes the main differences between the presented machine learning approaches: synchronous and asynchronous. Although the synchronous BCI systems are easier to design and evaluate, they offer less degrees of freedom to the user due to their cue-based nature.

**Table 2:** Comparative summary of two approaches used in BCIs nowadays.

ML Approach	Advantages	Drawbacks
**Synchronous BCIs**	Easier control for user artifacts: user has predefined time slots to move/blink his/her eyes Easier design (system knows at which moment of time the command from the user will be received)	Commands are imposed by the system, user cannot decide when he/she performs an action
**Asynchronous BCIs**	Can be operated on free will of the user	Could be prone to the artifacts generated by the user (eye blinks and movements) Computationally more demanding as provides continuous classification in real-time

### Abstraction Level 3: Neural Mechanisms behind EEG-based BCIs and Paradigms or What are we able to detect?

There are several particular types of neural mechanisms that are widely used in BCIs and we will explain their basic functioning.

BCI paradigm is usually referred to as a mean of extracting a control signal. In this paper we would like to clarify the vocabulary of using the word “paradigm”: in most papers about BCIs as well as PhD manuscripts the authors refer to BCI paradigms as they discuss the underlying neural mechanisms and the activity that causes the neural mechanisms. Sometimes they also use the phrase “brain patterns”. We find that this could be confusing for someone who just started learning about BCIs. That’s why in our work we will use two different terms, one to describe the underlying brain activity directly, “neural mechanisms”, and the other term, “BCI paradigms”, to describe this activity that causes these neural mechanisms. The main neural mechanisms interpreted by EEG-based BCIs that are used nowadays, are the following:

- **Event Related De/Synchronization (ERD/ERS)**,
- **Event-Related Potentials (ERPs)**,
- **Steady State Evoked Potentials (SSEP)**.

We will now present a short summary of these mechanisms as well as how can we cause the brain activity that causes these neural mechanisms, the “BCI paradigms”.

1. **Event related de/synchronization (ERD/ERS)** indicates changes in rhythmic activity of the brain, often within 8–12 Hz. These changes are observed over primary sensory or motor cortical areas. The synchronization (ERS, increase in brain rhythm) occurs while processing sensory information or producing motor output. The desynchronization (ERD, decrease in brain rhythm) occurs while performing the movements, movement (motor) imagery or movement preparation. *Motor imagery* (MI) is one of the well studied *paradigms* used in BCI applications nowadays. During MI the user imagines moving various body parts, e.g. hands, feet, tongue [23]. Actual motor activity is not necessary, merely imagining or mentally rehearsing a motor activity generates similar activations as real movement. This makes paradigms such as MI possible. The location of the activations depends on the limb involved in the motor activity or imagined motor activity, given that each limb is mapped to a different location of the sensory motor cortex.
2. **Event-Related Potentials (ERPs).** Most neural mechanisms that are used in BCIs are based on event-related potentials (ERPs), an activation in a certain area of the brain in response to a stimulus (event). In general, event related potentials are either positive or negative (positive or negative amplitude of the potential). The name given to ERPs often starts by P or N depending on whether they are positive or negative, followed by a number in milliseconds that characterizes how much time the potential appears after the stimulus. Examples of ERP-based systems include:
  - **P300.** P300 is a positive action potential generated around 300 ms after the user makes a choice (conscious or otherwise). To elicit a P300 potential BCI systems generally use a visual or auditory stimulus that is presented in an “odd-ball” paradigm: a random sequence of target and non-target stimuli is presented to the user. The P300 associated to the presentation of the target stimulus is higher than the one associated with non-target stimuli [24].
  - **Error-related potentials (ErrP).** These systems exploit error related negativity (ERN), ERPs that are negative activations generated in the brain 150 ms after the stimulation onset when the user commits and error (even if not consciously aware) or when negative feedback is received [4]. ERNs are often used to produce adaptive BCI systems that can detect when the user perceives a classification error and adapt the classifier in accordance [25]. They also have important applications in interaction design, where they can serve to detect desirable properties of interactive processes, without explicit feedback from users [26].
  - **N400**: is another category of error related potentials that manifest as a negative activation 400ms after the stimulus onset. N400 is part of the brain response to words that are semantically deviant (“I would like to have a coffee with boots”, N400 will be produced as a reaction the the word “boots”). Some other types of stimuli like audio ones could also elicit N400 response. [27].
3. **Steady State Evoked Potentials (SSEP).** Stimulus is presented repetitively at high rates so that the implicated cortex of the brain cannot return to its resting state. There are several types of SSEP depending on the location: visual (VEP), auditory (AEP), and somatosensory (SEP). Steady State Visually Evoked Potentials (SSVEP) are very often used in BCIs: the user looks at a target flickering at a certain frequency, e.g. 15Hz. This causes a rapid succession of action potentials in the visual cortex, some of which are at the same frequency as the stimulation [1]. For instance, in a standard synchronous setting, SSVEP systems can achieve performances of around 83% in classification accuracy [28, 29]. Asynchronous SSVEP systems in such works as [30] can achieve anywhere between 67 (for 1s trials) and 91% (for 4s trials). Hybrid systems combining both SSVEP and another control modality (Eye-tracking, Motor Imagery) can improve the performance of SSVEP alone [31].

Table 3 summarizes the main differences between the presented neural paradigms and compares them based on the classification accuracies that are observed in some related work publications. Although we provide the classification accuracies in Table 3, those should be taken with precaution as we provide average percentages here, otherwise due to differences in the number of classes, training time, trial length, the accuracy cannot be used as stand-alone measure to compare different neural mechanisms and systems that use them.

**Table 3:** Summary of advantages and drawbacks of different neural mechanisms. Each of three main neural mechanisms is present with one of the examples. Comparison of different neural mechanisms based on the classification accuracies and training time.

Neural mechanism	Nature	Advantages	Drawbacks	Synchronous	Asynchronous	Training Time
**Motor Imagery**	ERD/ERS	Does not require any external stimulation Can be operated on free will of the user	Requires training	2 classes 72–96% Random< 70%	4 classes 65–75% Random < 55%	ML 10–30 min OC 1–2 months
**P300**	ERPs	Almost no training needed	Requires external stimulation Could provoke tiredness in users	6*6 symbol matrix 80% after 5 repetitions (xDawn)	95%	10 min at most
**SSVEP**	SSEP	Almost no training needed			95%	10 min at most

Though ERP, ERD/ERS, and SSEP are the most widely used neural mechanisms in most BCI systems, however they are not restricted to. Going further, there is an existing recent work that extends BCIs by use of self-regulation of a variety of different neural mechanisms beyond convention [32, 33, 34].

### Abstraction Level 4: Applications. Where and how can we use BCIs nowadays?

A lot of non-specialists in BCIs think that BCIs are used mostly as an assistive technology, but there are quite a few applications for BCI-based systems that integrate this technology.

**Fig 3:**
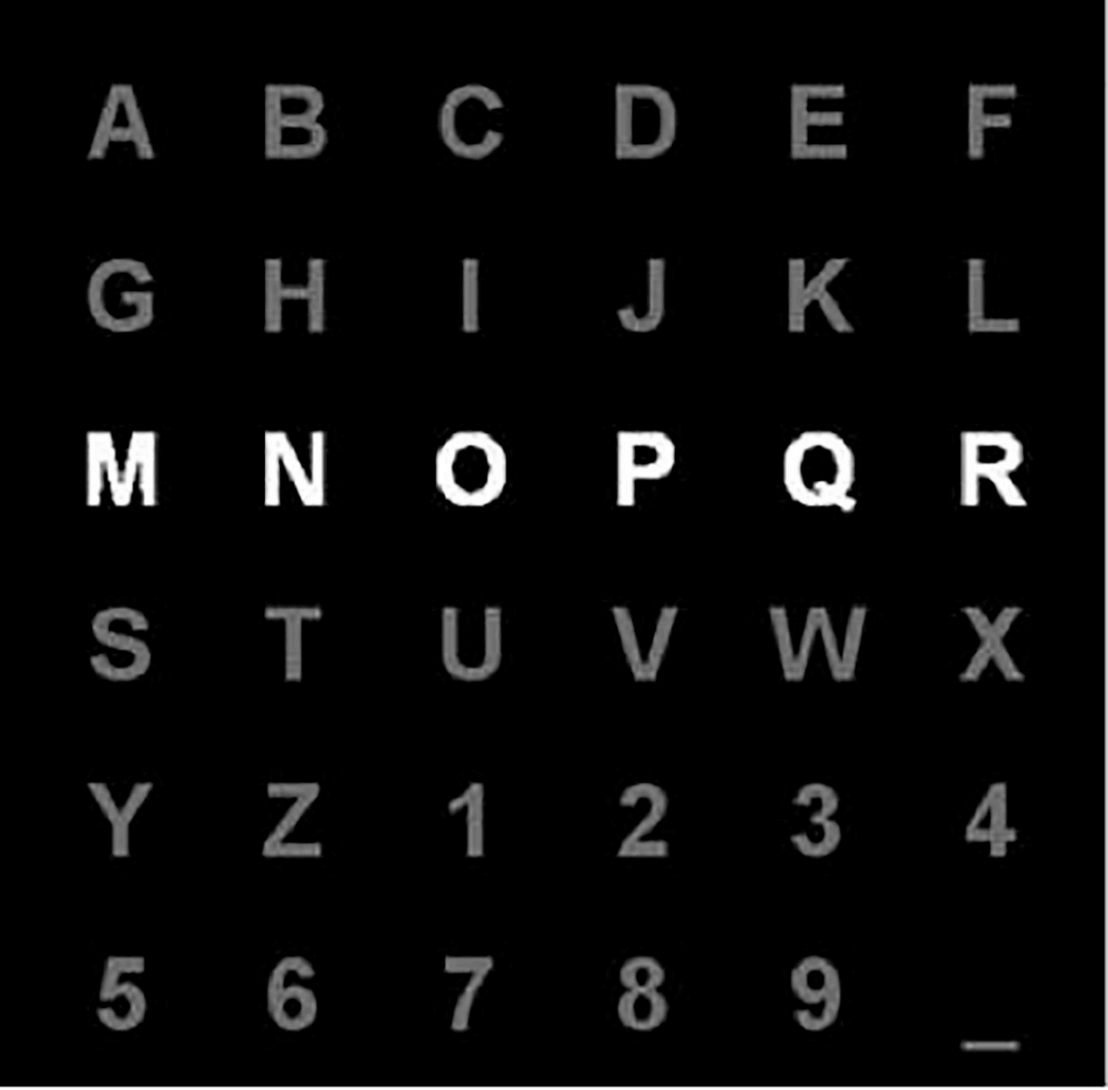
A matrix which contains letters of the alphabet and other symbols, which are flashed in random order (white line on the image) to elicit the P300 evoked response based on [35].

Up till now we discussed the tools we need to use in order to acquire the brain activity, what information can we acquire and how can we analyze it. Let’s now see the concrete examples of the applications we are actually having nowadays. We analyzed around 100 papers about BCIs from 1985 till 2016, and regrouped the applications into the following 8 categories:

1. **Communication.** Usually referred to as yes/no communication, one of the first applications where BCIs were used. A famous example is a system called “Right Justified Box”, in which motor imagery was used to choose one of two targets [35].
2. **Typing.** Typing is the second oldest application for BCIs and is one of the most common applications developed and used nowadays in BCI industry. One of the most extensively studied approaches is “Farwell-Donchin Matrix” [36]. The matrix is composed of letters of the alphabet and other symbols that are flashed in random order in order to measure the P300 evoked response (Fig 3). Nowadays the systems that use “Farwell-Donchin Matrix” require very minimum to no training and can achieve up to 100% of classification accuracy.
3. **Web surfing.** Moving further from typing to the control of the whole web browser was proposed by several research groups. For example, “The Brain Browser” by [37] was based on motor imagery to choose the commands such as “next” and “previous”.
4. **Manipulating.** In this category of applications we refer to the applications that are used to directly manipulate (change speed, send a command to turn left) the virtual and/or physical objects, e.g. moving forward a wheelchair, selecting an item in a video game and so on. Here are two examples:
  - *Real robot piloting task*. From [38]. A user is able to control a real robot drone by performing mentally the following tasks: rising it up by imagining both hands movement; going down by imagining both feet movement and so on. The system achieved around 95% accuracy after 2 months training period. Other examples of robot control include [39].
  - *Virtual apartment control*. [40] introduced a system to control a virtual apartment, where the possible commands and actions were presented in the form were presented on a screen in the form of “Farwell-Donchin Matrix” and the borders of the images were flashed to elicit the P300 evoked response. The system achieved 95% accuracy with 4 different options.
5. **Human-Aided Computing.** This term was first proposed by [41] in order to describe the systems that use in their decision making the results from the implicit processing that the human brain already performs (e.g. a human observes a candle, it does not require any particular mental action associated with this task, but the brain identifies and labels the candle automatically just by passively viewing it). Although the machine learning tools are considered as very powerful nowadays, the human brain is more efficient in, for example, labeling the data in the environment. Thus, we can help the existing pattern recognition systems to recognize and label the images of other stimuli more efficiently and faster.
6. **Creativity applications.** [42] proposed a system, that generates music based on the dominant frequencies from EEG signals. The output of the music engine was influenced by the output of the detected dominant frequency at the moment of time.
7. **Health-related applications.** The applications are also very numerous within this domain, as BCIs were initially proposed as a solution for people with disabilities. The applications include coma detection (detecting the presence of cognitive function, [43]; treatment for Attention Deficit Hyperactivity Disorder (ADHD, [44]); rehabilitation and prosthetics, including the therapies to restore motor control after stroke [45]. Given that one of the ultimate motivations of BCI was to exploit restoration of motor control in stroke [46, 47], significant progress has been made in the past years regarding that [48, 49, 50]. Most applications for rehabilitation use “manipulating”-based systems, described previously, as well as appropriate feedback strategies, for example, visual or auditory feedback for providing a patient with the guidelines on his/her progression. Examples include [50] where patients after a stroke were instructed to perform a motor imagery of their affected hand but they also received a visual feedback of a virtual hand moving which was interimposed on their own.
8. **Cognitive state monitoring applications.** The examples here include any potentially safety-critical jobs, where the intense level of concentration of a human is needed, for example in air-traffic control but also in any applications for better UX, e.g. modifying the layout of the webpage if the system perceives that the user is overwhelmed with tasks. In physical environment one example may include the light change and music switch in the apartment, if the person is perceived as tired. Here we cite five examples:
  - *Reading engagement application* from [51], where a user is reading a text, and at some moment of time the text is perceived by the user as boring (measured with a BCI), a video related to current text will appear, in order to attract the attention of the user to what he/she is reading.
  - [3] detected the periods of *boredom or overload in order* to adapt the task to the user from one moment of time to another. In their experiment participants performed path planning task for multiple unmanned aerial vehicles (UAVs) in a simulation. Based on their mental state, the difficulty of the task varied by adding or removing UAVs and the authors found that it was possible to decrease errors by 35% over a baseline condition.
  - *Notification systems*. Phylter system by [52] used the cognitive state of the user and the information delivered by the user in order to decide whether or not to deliver the notification message depending on the message’s specified priority and prediction about the user’s interruptibility.
  - *Meditation training*. [53] proposed a study with a group of subjects performing meditation and a control group of subjects, who were not performing this task. The subjects performing the meditation, showed a presence of an ERD of beta rhythm during the resting state. This ERD was not found in the control group.
  - BCIs as *a tool for accessing UX*. [54] proposed to use EEG-based BCIs as an evaluation tool during HCI experiments, by accessing user’s mental workload, attention and recognition of interaction errors.

These examples illustrate the great variety of the systems that use BCI: the different applications: from manipulating a drone in physical environment to the changes in the interface to adapt to the workload level of the users; the different ways of achieving the control over the system (imagining a movement vs. not performing any particular action), the variety of the platforms the BCIs are used in (real robots vs. virtual environment). How can we take this great diversity of systems and meaningfully reason about them and design within their space? To answer this question, we first present a review of the taxonomies from both HCI and BCI, to see how the researchers reason about them up till now.

## Abstraction Level 5: Introducing a conceptual space for EEG-based BCIs

Our conceptual space contains 4 axes which represent 4 questions: *When* (temporal aspects), *What* (content aspects), *How* (medium aspects) and *Where* (spatial aspects). These 4 axes contain 9 sub-axes. The conceptual space consists of 41 options in total as well as their different combinations.

Fig 4 shows our conceptual space. We now present each axes in more details.

**Fig 4:**
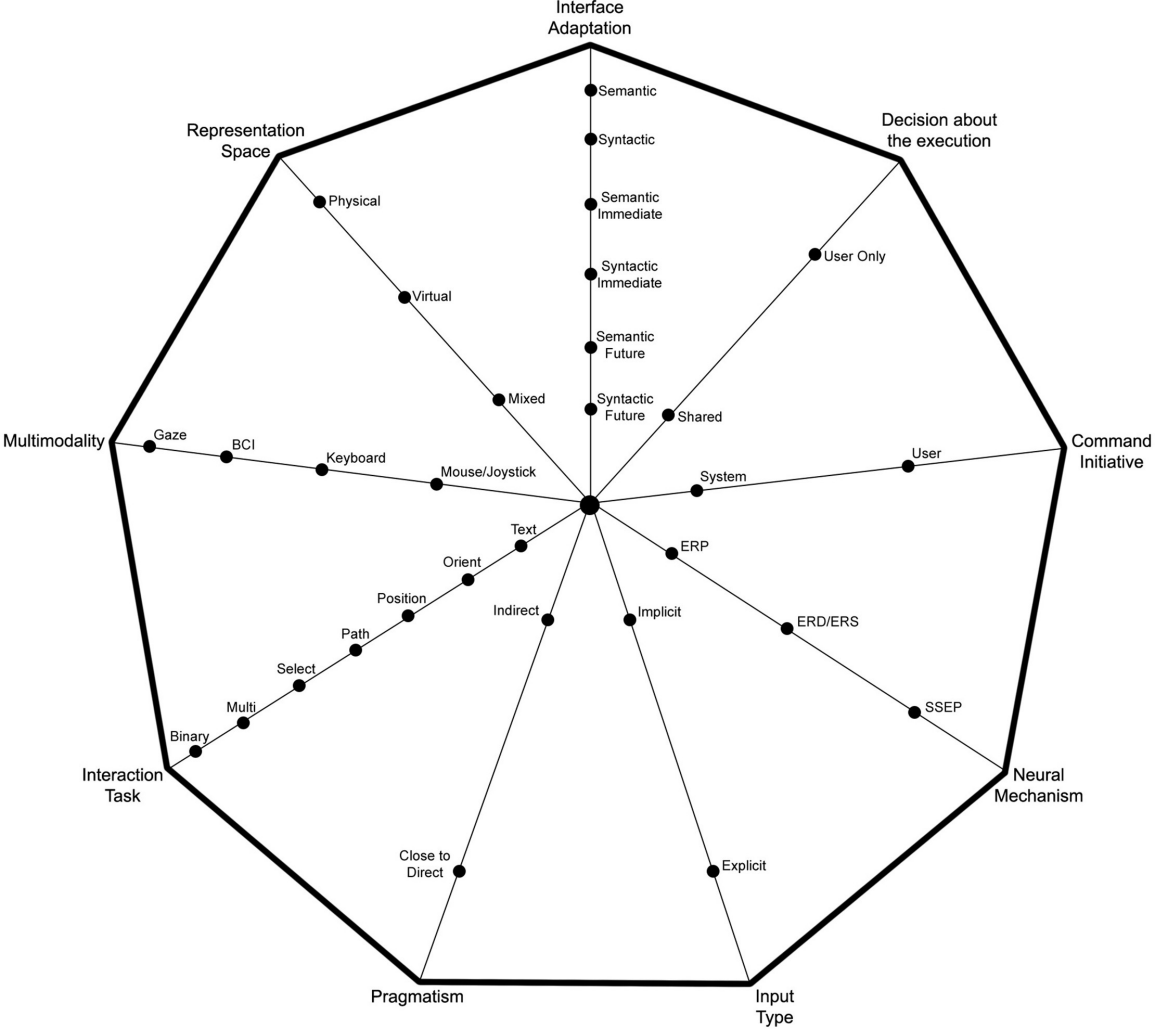
Our conceptual space of EEG-based BCI applications.

### Axis when

This axis characterizes the notions related to temporal features of BCIs. As we consider the execution of one BCI command as the referent unit of the time, thus we distinguish the beginning of the command (i.e trigger) and its execution.

We define three aspects (sub-axes) describing this axis:

#### Input type

Here we distinguish *explicit* and *implicit* input. When the user consciously produces a mental action, the input is considered as being explicit. In contrast, *implicit input indicates* cognitive changes of the user, that the system takes into consideration “but that were not actively chosen by the user to interact with the system” [55]. For example, the “Alpha WoW” system integrated a BCI to the “World of Warcraft” game [56]. The BCI was used to evaluate the players stress level, and changed accordingly the appearance of its avatar from an elf to a bear (Fig 5).

**Fig 5:**
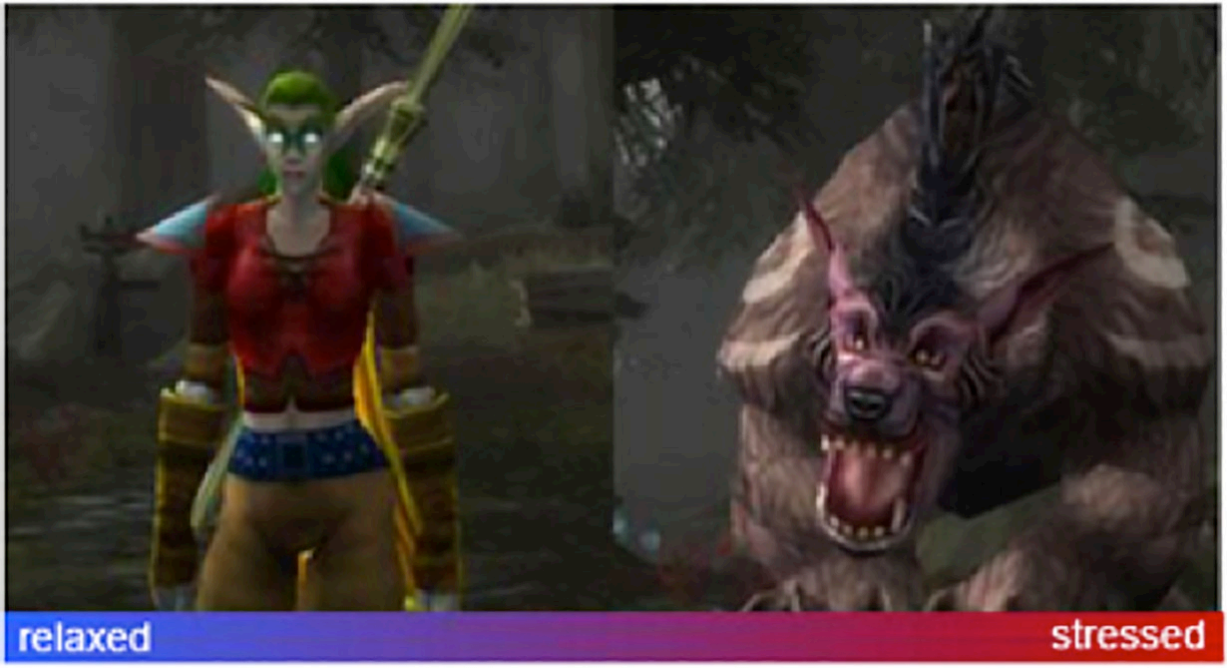
“Alpha WoW” application where a user avatar can transform itself in a bear depending on the users stress level [56].

The input type sub-axis is inspired by one of the most cited classifications in the state of the art literature proposes separating them into active, reactive and passive BCIs [57]. *Passive BCIs* monitor and detect the state of the user (e.g. workload monitoring); *reactive* in which external stimuli are presented to the user and in which the resulting activations are recognized (mostly for speller applications); *active*, in which users voluntarily perform an imagined mental action and in which the resulting activity is recognized (e.g. direct control applications).

For our current version of the framework we simplify the proposed classification and only retain “active/passive” notion. By simplifying the proposed classification we consider that the mental activity that is obtained from the user could be either *implicit* and he/she is not doing ANY effort and potentially not even aware about the type of the input he/she is giving to the system, either *explicit*, when the user replies to the stimuli, performs any type of imagination or needs to pay the attention towards something.

#### Command initiative

BCIs could be either: system-paced, in which the timing of the commands is imposed by the system, or self-paced, in which commands are issued at any time the user decides. We thus distinguish an *initiative* coming from a *system* as in the first case, or *initiative* coming from a *user*, as in a second case. This command initiative axis represents a *synchronous/asynchronous* training phase presented from previous section.

#### Decision about the execution

Decision about the execution of the command “could either rely on *user only* or it could be executed in a *shared* manner, meaning that the mental action from the user is transformed into a high-level command that is sent to the application” [10]. For example in [58] a user controls a wheelchair by selecting a destination using P300, and then a decision-making program decides about the final movement, taking into consideration the additional information from the other sensors about the obstacles.

### Axis what

This axis represents the information conveyed by BCI and for what it has being conveyed (execution of which tasks?).

We define three aspects describing this axis:

#### Neural Mechanism

As we discussed in previous section of this paper, we distinguish the following neural mechanisms that are mostly interpreted by BCIs nowadays: ERD/ERS, SSEP, ERP.

#### Interaction Task

We re-use Foley’s interaction tasks for our conceptual space: Select, Position, Orient, Path, and Text [59]. We also add a trigger task (on/off, yes/no) that is common in BCIs (see “communication” applicative domain in this paper as well as [60]), as well as the « multitasks », which stands for of the six aforementioned interaction tasks of Foley’s taxonomy. Although these tasks are nowadays used in most of BCI-based systems, we also introduce a notion “N/A” which stands for the systems, which do not present the tasks. In the example of Fig 2, moving a wheelchair to the left by imagining left hand movement corresponds to the “orient” task.

#### Pragmatism

We introduce a *pragmatic property* in our conceptual space, to highlight the directness of the “mapping between the user’s expectation (i.e. goal) and the semantics of the interaction technique carried by the computer” [61, 62].

As an example we can take an imagination of the feet movement within two different tasks: in the task of lifting a virtual spaceship [63] and navigating through the virtual museum [64]. As navigating is related to walking, a task of imagining feet movement to perform the action is considered by us as *a direct pragmatic mapping*. But we usually do not imagine/use feet to lift an object except some games like football, so asking to imagine the feet movement in the case of spaceship lifting is *not a direct mapping*. The pragmatic axis is important as the lack of logical meaning in the mapping may impact the performance of the task drastically [65].

### Axis how

This axis represents how the system conveys the information from BCI in relation with other modalities (if any) available along with BCI.

We define two aspects describing this axis:

#### Multimodality

By multimodal concept in this paper we refer to the “hybrid” concept introduced by Zander and Pfurtscheller. BCIs are usually combined with the following input devices: “a keyboard [66], a computer mouse [67], or a joystick [68]” [10]. Several types of BCIs were combined together [69; 70]. The BCI system was also combined with different input modalities, such as a speech recognition system [71] or “eye-tracking, for instance, using P300 [72] and motor imagery [57] BCIs” [10]. In our conceptual space option “Yes” means that another system (either “Gaze”, “BCI”, “keyboard”, “mouse”) was used in conjunction with the BCI and “No” means that no other system was used. If we consider an EEG-fNIRS BCI system as in [73] we indicate “Yes” in “Multimodal Axis” and then we will select a “BCI” option among the four alternatives for the additional interaction option. We highlight the importance of these axes, as other inputs (e.g. gaze) could help overcome the complexity of signal processing and classification (steps 2 and 3 in a typical BCI system described earlier).

#### Interface adaptation

The interface could be *adaptive (“yes”* option in the axis), meaning that it is able to adapt itself to a change of the current context of use (user, platform, environment) [72] or *non-adaptive* (*“no”* option in the axis), where the context of use is predefined in advance and is not changing. If the “yes” option is selected, we then rely on the proposed framework of [74]: “adaptations are categorized by their target functional level; *semantic* or *syntactic*, and immediacy: *immediate* or *future* changes”.

### Axis where

#### This axis covers only one aspect–representation space

It shows the objects with which the user interacts. The user could manipulate the physical objects, or the virtual ones (numerical). Between these two extremes there is also the possibility that the user is manipulating a physical object but augments its properties with the additional numeric information (mixed). An example of manipulating the physical objects includes the “*Real robot piloting task”* from [21], where a user is able to control a real robot drone by performing mentally the following tasks: rise up; by imagining hand movement; go down; by imagining feet movement and so on. An example of manipulating virtual objects would include “*Virtual apartment control”* from [40], where users were selecting the images on the screen to manipulate the corresponding virtual objects in the apartment.

## Classification of existing BCI systems

### Evaluation of conceptual space

In order to evaluate our conceptual space, we consider the three dimensions proposed by [62] to evaluate a design space:

1. *descriptive power*: “the ability of the design space to describe a significant range of existing interfaces”;
2. *evaluative power*: “the ability of the design space to help assess multiple design alternatives”;
3. *generative power*: “the ability of the design space to help designers create new designs”.

The fullness of the conceptual space cannot be definitely proven, but we propose two solutions that facilitate the description and evaluation of our conceptual space. First of all, we have chosen one system from each of eight applicative domains presented in section “Abstraction Level 4” of this paper, in order to address the descriptive power of our conceptual space. We then have chosen to zoom into two of the domains in more depth and describe 32 state of the art systems coming from two applicative groups: house control and games. For our gaming analysis we inspired ourselves with the table from chapter 10 of [65], where 37 games based on BCIs are classified regarding which paradigm was used, which type of sensors, how many commands were achieved and with which level of accuracy. The authors point out that “due to differences in the number of classes and trial lengths, these accuracies cannot be used to compare individual studies”. As for the second solution, it could be complicated to find some unexplored zone in our conceptual space, when more systems will be added; as well as classification and evaluation of the new systems that would be added to the conceptual space may not be an easy task. In order to facilitate the usage of our conceptual space, we will propose an interactive webpage, where we describe the functionalities of our conceptual space, and where the other researchers will be capable to add the new systems or visualize the systems they would like to compare.

Finally, the generative power of our conceptual space will be discussed in details in the following section, with the conception and experimental evaluation of the two new interaction systems.

To illustrate the descriptive power of our conceptual space, we will now project several existing BCI systems in Tables 4, 5, 6 and 7. Table 4 includes the systems from the 8 applicative domains we described in Section “Abstraction Level 4” of this paper. We take one system from each application domain and project it in our conceptual space in order to address the descriptive power of our conceptual space. We then have chosen to take the BCI systems within two domains (home control, Table 5; and gaming, Tables 6 and 7) in order to check, if some tendencies could be seen if the systems are analyzed via one domain. The cross (“X”) in the tables indicates the presence of the feature. Each axis could contain as many features as there are in each of the projected systems. For example, if we consider an SSVEP-ERD system of [75], where alpha-ERD is used to change avatar speed while SSVEP is used to destroy the enemy, we will select ERD and SSEP in “Neural mechanism axis”. We leave the empty lines in the tables on purpose, for better illustrating the future research directions and applications of this study in the following section on future work.

**Table 4:** Classification of EEG-based BCI applications within our conceptual space from 8 different applicative domains identified in section “Abstraction Level 4” of this paper.

	Communication, [35]	Typing, [36]	Web surf, [37]	Manipulating, [38]	Human-aided computing[41]	Creativity, [42]	Health, [43]	Cog.state [54]
**Rep.space :**								
Physical				X		X		
Mixed								
Virtual (Numerical)	X	X	X	X	X	X	X	X
**Neural Mechanism :**								
SSEP								
ERD/ERS	X		X	X		X	X	
ERPs		X			X		X	X
**Interaction task :**								
On/off						X		
Yes/no							X	
Path								
Text		X						
Orient				X				
Position	X		X					
Select							X	
Multi								
N/A					X	X		X
**Pragmatism :**								
Indirect							X	
Close to direct	X	X	X	X				
N/A					X	X		X
**Multimodality :**								
Yes								
No	X	X	X	X	X	X	X	X
*Complimentary input device (if yes) *:								
Gaze								
BCI								
Mouse/joystick								
Keyboard								
**Interface adaptation :**								
Yes								
No	X	X	X	X	X	X	X	X
*Adaptation functional level (if yes) *:								
Semantic								
Syntactic								
*Immediacy of adaptation *:								
Semantic Immediate								
Semantic future								
Syntactic Immediate								
Syntactic future								
**Decision about the execution :**								
User-only	X	X	X	X	X	X	X	X
Shared								
**Command initiative :**								
User-triggered	X			X		X		X
System-triggered	X	X	X	X	X	X	X	
**Input type :**								
Explicit	X	X	X	X			X	
Implicit					X	X		X

**Table 5:** Classification of EEG-based BCI applications for smart homes within our conceptual space.

	[40]	[76]	[77]	[78]	[79]	[80]	[81]
**Rep.space :**							
Physical							
Mixed							
Virtual (Numerical)	X	X	X	X	X	X	X
**Neural Mechanism :**							
SSEP				X			
ERD/ERS		X					
ERP	X		X	X	X	X	X
**Interaction task :**							
On/off			X		X	X	X
Yes/no							X
Path					X		
Text							
Orient							
Position							
Select	X		X	X			
Multi		X					
N/A							
**Pragmatism :**							
Indirect					X	X	X
Close to direct	X	X	X	X			
N/A							
**Multimodality :**							
Yes		X		X	X		
No	X		X			X	X
*Complimentary input device (if yes) *:							
Gaze		X					
BCI				X	X		
Mouse/joystick							
Keyboard							
**Interface adaptation :**							
Yes							
No	X	X	X	X	X	X	X
*Adaptation functional level (if yes) *:							
Semantic							
Syntactic							
*Immediacy of adaptation *:							
Semantic Immediate							
Semantic future							
Syntactic Immediate							
Syntactic future							
**Decision about the execution :**							
User-only	X	X	X	X	X	X	X
Shared							
**Command initiative :**							
User-triggered							
System-triggered	X	X	X	X	X	X	X
**Input type :**							
Explicit	X	X	X	X	X	X	X
Implicit							

**Table 6:** Classification of studies that use games (both physical and virtual) using EEG-based BCI applications within our conceptual space (part 1).

	[82]	[83]	[84]	[85]	[86]	[87]	[88]	[89]
**Rep.space :**								
Physical		X						
Mixed								
Virtual (numerical)	X		X	X	X	X	X	X
**Neural Paradigm :**								
ERP					X	X		X
SSEP				X			X	
ERD/ERS	X	X	X					
**Interaction task :**								
On/off				X				
Yes/no								
Path								
Text								
Orient								
Position		X	X				X	
Select				X		X		X
Multi								
N/A								
**Pragmatism:**								
Indirect			X		X	X	X	
Close to direct				X				X
N/A	X	X						
**Multimodality :**								
Yes	X							
No		X	X	X	X	X	X	X
*Complimentary input device (if yes) *:								
Gaze								
BCI								
Mouse/joystick	X							
Keyboard								
**Interface adaptation :**								
Yes	X							
No			X	X	X	X	X	X
*Adaptation functional level (if yes) *:								
Semantic	X							
Syntactic								
*Immediacy of adaptation *:								
Semantic Immediate	X							
Semantic future								
Syntactic Immediate								
Syntactic future								
**Decision about the execution :**								
User-only		X	X	X	X	X	X	X
Shared	X							
**Command initiative :**								
User-triggered		X	X			No info	X	X
System-triggered	X		X (for evaluation)	X	X	No info		X
**Input type :**								
Explicit		X	X	X	X	X	X	X
Implicit	X							

**Table 7:** Classification of studies that use games (both physical and virtual) using EEG-based BCI applications within our conceptual space (part 2).

	[90]	[91]	[92]	[93]	[94]	[95]	[96]	[97]	[98]
**Rep.space :**									
Physical	X								
Mixed									
Virtual (Numerical)		X	X	X	X	X	X	X	X
**Neural Paradigm :**									
ERP									
SSEP									
ERD/ERS	X	X	X	X	X	X	X	X	X
**Interaction task :**									
Yes/no						X			
On/off (trigger)									
Path				X	X			X	
Text									
Orient	X	X	X				X		
Position									X
Select									
Multi									
N/A									
**Pragmatism :**									
Indirect					X	X	X		
Close to direct	X	X	X	X				X	X
N/A									
**Multimodality:**									
Yes									
No	X	X	X	X	X	X	X	X	X
*Complimentary input device (if yes) *:									
Gaze									
BCI									
Mouse/joystick									
Keyboard									
**Interface adaptation :**									
Yes									
No	X	X	X	X	X	X	X	X	X
*Adaptation functional level (if yes) *:									
Semantic									
Syntactic									
*Immediacy of adaptation *:									
Semantic Immediate									
Semantic future									
Syntactic Immediate									
Syntactic future									
**Decision about the execution :**									
User-only	X	X	X	X	X	X	X	X	X
Shared									
**Command initiative :**									
User-triggered	X							X	X
System-triggered	X	X	X	X	X	X	X		
**Input type :**									
Explicit	X	X	X	X	X	X		X	X
Implicit									

### Usage scenarios

Here we illustrate how our conceptual space could be useful for the users who would like to find the unexplored research axes–for instance, the researchers in HCI.

#### Scenario 1: Fast preview of existing systems

John, a PhD student in HCI would like to see some generic tools of the existing BCI systems. Although some domains concerned by BCIs such as signal processing, propose the state of the art papers about the classification algorithms like [99, 100], no works exist to the general classification of BCI systems. By using our tool, John gets a starting point of the list of the BCI systems. After reading the proposed references, John forgets some details or does not see any particular difference between some systems, but our conceptual space helps him to see all the systems at a glance. The supervisors of John work in ambient intelligence and suggest him to investigate the works related to home control, as the PhD of John is made in partnership with a company, working on smart sensors for the apartments. John selects two works of [78] and [79] suitable for his project. He wants to further investigate the main difference between the works of [78] and [79], and he discovers that Edlinger *et al*. are using 2 types of BCI systems, otherwise the systems are very similar, and John finds the implementation of Edlinger more beneficial, as it uses 2 types of BCIs and its pragmatism is close to direct.

#### Scenario 2: Creating a new system

Jane, an HCI researcher, decided to explore the usage of BCIs. Using our conceptual space, she is able to find the unexplored zones in it. It shows her that there are not a lot of systems that apply BCIs in physical space, and no system actually proposes a mixed interaction. Jane considers that it could be of interest, as nowadays BCIs are still considered as additional interaction techniques that are not reliable enough to be used for critical control, and using BCIs to provide some additional information in an augmented reality is potentially an application to be tested. If Jane wants to move on, by checking the adaptive interfaces, she will be able to notice, that no system is proposed today that uses syntactic future changes. Moreover, in HCI, adaptation to the user needs is an important property for some systems. She checks the reference of the study that initially proposed the theoretical concept of this approach and considers the further investigation of this axis in her own study.

### Further empirical validation of conceptual space

In order to further validate our conceptual space, we performed a focus study with 8 HCI researchers, who all has 5+ years of research experience and publishing in top HCI journals and conferences. We asked them to look through the proposed document as well as the matrix from Fig 4 and Tables 5 to 8 and to come up with a new system for BCIs that would fill in the gap and preferentially would build on previous, existing works and systems. The researchers focused on the smart home applications and they proposed a new system based on [76] with the following reasoning: “*we have already known that BCIs are error-prone systems but using the proposed conceptual space we have discovered that a lot of works couple BCIs with gaze tracking. As we focused on smart home applications, we have found out that [76] uses gaze to select an object in the house to be controlled, and then suggests imagining an action to be performed with an object. We were intrigued that no other system uses gaze and a “simpler” to be trained BCI, like SSEP. Although flickering might not be the most pleasant interaction, it seems to be a more robust way of communication based on BCIs than using ERD/ERS. Moreover, in the use case of the home control, there are tens of objects to be controlled and thus SSEP will be easier to control and to use than ERD/ERS*”.

**Table 8:** 11 CHI papers from 2010–2016 within our conceptual space.

	[51]	[103]	[104]	[105]	[106]	[107]	[3]	[108]	[109]
**Rep.space :**									
Physical		X	X	X				X	
Mixte									
Virtual (Numerical)	X				X	X	X		X
**Acquisition:**									
(f)NIRS					X		X	X	X
EEG	X	X	X	X		X			
ECoG									
(f)MRI									
PET									
MEG									
**Neural Paradigm :**									
ERP				X					
SSEP									
ERD/ERS	X	X	X						
**Interaction task :**									
On/off			X						
Yes/no									
Path									
Text									
Orient									
Position									
Select				X					
Multi									
N/A									
**Pragmatism:**									
Indirect			X						
Close to direct				X	X		X	X	X
N/A	X	X				X			
**Multimodality:**									
Yes									
No	X	X	X	X	X	X	X	X	X
*Complimentary input device (if yes) *:									
Gaze									
BCI									
Mouse/joystick									
Keyboard									
**Interface adaptation:**									
Yes	X				X	X	X	X	X
No			X	X					
*Adaptation functional level (if yes) *:									
Semantic	X				X	X			X
Syntactic		X					X	X	
*Immediacy of adaptation*:									
Semantic Immediate	X				X	X			
Semantic future									X
Syntactic Immediate		X					X	X	
Syntactic future									
**Decision about the execution :**									
User-only			X	X					
Shared									
N/A	X	X			X	X	X	X	X
**Command initiative :**									
User-triggered			X						
System-triggered	X	X		X	X	X	X	X	X
**Input type :**									
Explicit			X	X					
Implicit	X	X			X	X	X	X	X

## Discussion, limitations and future work

### General discussion and limitations of conceptual space

The analysis of the conceptual space by representing 32 BCI systems reveals several interesting facts: the majority of the systems are applied and used in *virtual (numerical space)*, most of the systems use *ERD/ERS as neural paradigm*, and in most of the cases the user needs to wait *the system trigger* to start any mental activity.

#### ERD/ERS as neural paradigm

This is an interesting finding, as ERP/SSEP paradigms tend to show almost no training needed and it is possible to achieve 100% accuracy with those. This finding could be explained by the convenience of use of ERD/ERS paradigms, as they do not require any additional stimuli in the environment to be measured.

#### The system trigger

BCIs that use system triggers, only takes into consideration the brain activity during predefined time windows. Therefore, the user can manipulate the system only during these predefined time windows. The advantage of such BCI systems is in their relative robustness, as the system knows in advance the type of activity to be expected from the user and the precise moment of time this activity could happen. This simplifies the design and evaluation of such systems. These systems are computationally less demanding and complex, but they are also restricting the interaction between the user and the system.

More generally, there are some tendencies within the sub-axes. For example, if we choose the “Interaction Task” we can see the clear difference for each of the tables: the most common tasks for smart homes are “on/off”, “position” and “select”; for the games–“path”, “orient”. These trends exist through the whole conceptual space within each axis.

In addition to these main observations, the conceptual space could be used as a tool to understand intrinsic differences even among very similar systems such as, for example, between [82] and [83] which both look very similar at the first sight but one system uses hybrid interaction and not the other one. Moreover, as we can notice, in the case of smart home control, several systems use hybrid interaction in conjunction with the BCIs mostly for selection task, which is not the case for the gaming domain, where almost no systems use hybrid interaction in conjunction with the BCIs.

The conceptual space proposes a first state of the art classification framework, where each system is represented with the respect of already existing BCI taxonomies but also applying the HCI considerations to the input devices. Typically, reference BCI taxonomies such as [56] do not consider the command initiative (cf. user-triggered or system-triggered), nor do they cover the interaction space (physical or virtual). The proposed approach allows the classification of a wider range of interaction techniques that could be presented in future. Moreover, it considers a fine grain analysis of the implemented user control (cf. the decision about the command, command initiative and pragmatic axes) allowing the unique integration of original interaction techniques such as the one proposed by ourselves to show the generative power of the conceptual space.

The conceptual space can be also used as a framework for decision making about new applications. For example, by proposing an application, that uses a new neural paradigm for the tasks from Foley’s taxonomy while designing an application, the researchers will be able to test a new user experience with BCI.

We tried to include the axes that span across different levels of descriptions and come from different fields of expertise (BCI and HCI), as we consider that it actually provides the framework to merge the expertise of two different areas and to help in thinking “outside of the box”. We would like to point out, that also we grouped the 9 sub-axes into 4 axes, they could be seen as separate dimensions, as the importance of the axes and sub-axes depends on the end user of the framework (e.g. a BCI researcher or a HCI researcher) and on the aim of using the tool at first place (descriptive of the state of the art, or generative of new lines of research). As an example, we would like to cite one of the experts of the domain who reviewed our conceptual space: “for a BCI expert the sub-axis “Representation Space” might not be as important as the sub-axis “Interface Adaptation”. For a BCI developer/programmer, it might indeed be less important to know if the considered system is used for controlling a real-world object (e.g. a flying drone) or a virtual object (e.g. a cursor on the screen), as a BCI researcher might be rather interested in developing/optimizing the underlying algorithm for recognition of the different mental states, which would control the object independently of its nature. On the other hand, it might be more important for the same BCI researcher to classify the type of adaptation (i.e. the categorization “semantic” or “syntactic”, with “immediate” or “future changes”), to propose new adapting schemes not imagined before”.

The current paper does not take into account or explore by any mean the training and feedback in BCIs as there is a design space already published which tackle these aspects of BCI systems [10]. In our conceptual space the “classical” taxonomies for input devices in HCI have been used as a basis to provide researchers and designers from HCI with an overview of BCIs close to the one of the “classical” GUIs.

### Future work: Towards a conceptual space for any BCI applications?

Our conceptual space only takes into account EEG as an acquisition technique for BCI applications. But as we presented in section “Abstraction Level 1: Signal Acquisition” of this paper, we distinguish 5 more acquisition techniques that are mainly used nowadays: (f)NIRS, (f)MRI, ECoG, PET and MEG. We hypothesize that it could be possible to include the other acquisition methods as well. For instance, given that the phenomena measured by EEG and MEG are the same, the study of ERPs is similar, and thus we do not need to make any particular modifications in our conceptual space. We analyzed 9 papers from CHI proceedings and presented the results in Table 8, where the acquisition techniques that are often presented include fNIRS. For this we added “Acquisition Axis” to our conceptual space, and we did not do any further modifications to our conceptual space. We highlight the new axes in dark grey color. We have chosen CHI conference, as this work aims on facilitating the accessibility of BCIs for HCI at the first place. As we can see after projecting these studies on our conceptual space, for the papers published at CHI, there is a clear tendency towards the systems that use interface adaptation, so the mental activity that is demanded from the user is implicit.

Going further, as we have mentioned in section 2.1.3 about neural mechanisms, there is existing recent work that extends BCIs by use of self-regulation of a variety of different neural mechanisms beyond ERPs, ERD/ERS and SSEPs [32, 33, 34]. These recently developed BCI systems that are proposed as personalized communication and control channels for ALS patients, which could lie outside the current version of the proposed concept space in the neural mechanism dimension could be easily integrated in the “Neural Mechanism” axis. Moreover, several recent papers feature increased number of systems that use multimodal BCIs like EEG and MRI [101, 102] and thus, the current version of the design space can be further easily updated, if needed.

## Conclusion

Nowadays, the adoption of BCIs as an input modality is still quite moderate aside from more « proof-of-concept » applications. We argue that it is in part related to the fact that the BCIs and its commands are not self-revealing: both users and designers should know which commands are available and how to trigger them.

There is currently a gap as no design space on BCIs exists that would help the users, designers and researchers to better understand, compare and reason about the appropriate solutions the BCIs could provide to them. The challenge is to propose a design space, which would be simple to use by the researchers and users who may not be familiar with the full state of the art on BCIs.

In this paper we proposed a novel conceptual space that describes the EEG-based BCI systems. Our conceptual space contains 4 axes which represent 4 questions: *When* (temporal aspects), *What* (content aspects), *How* (medium aspects) and *Where* (spatial aspects). These 4 axes contain 9 sub-axes. The conceptual space consists of 41 options in total as well as their different combinations.

The analysis of the conceptual space by representing 32 existing BCI systems revealed several interesting facts: the majority of the systems are applied and used in *virtual environment*, most of the systems use *ERD/ERS as neural paradigm*, and in most of the cases the user needs to wait *the system trigger* to start any mental activity. In addition to these main observations, the conceptual space could be used as a tool to understand intrinsic differences even among very similar systems. The conceptual space can be also used as a framework for decision making about new applications.

Although our conceptual space is built around EEG-based BCIs, we further demonstrated a possibility to generalize our conceptual space to cover other acquisition techniques like fNIRS.

Brain-Computer Interfaces have received much attention over the last years. We believe this is because, at core, they are leaving the labs, and taking steps into the “real” world. We hope that our conceptual space will encourage the development of novel BCI applications, and make the fusion between BCIs and HCI more fluid.
